# Pneumothorax Identified by a Remote Physician Using Paramedic-obtained Tele-ultrasound: Case Report

**DOI:** 10.5811/cpcem.1296

**Published:** 2024-06-03

**Authors:** Shriman Balasubramanian, Michael DeFilippo, Michael Stone, Gabriela Galli, Matthew McCarty, Brock Daniels

**Affiliations:** * New York Presbyterian Hospital Cornell and Columbia, Department of Emergency Medicine, New York; † Weill Cornell Medicine, Department of Emergency Medicine, New York

**Keywords:** *telehealth*, *case report*, *community paramedicine*, *pneumothorax*, *point-of-care ultrasound*

## Abstract

**Introduction:**

The use of telemedicine and ultrasound is emerging and novel in the field of community paramedicine. However, there is a paucity of data supporting its use and even less evidence that shows a morbidity and mortality benefit. This case highlights a unique way to diagnose a common medical emergency, which can lead to a good outcome.

**Case Report:**

We describe the use of lung point-of-care ultrasound by a trained community paramedic that led to the identification of a pneumothorax in an 86-year-old male at a scheduled home visit. The images were interpreted over telehealth in real-time by an emergency physician, and the patient was transported to the emergency department where the diagnosis was confirmed by chest radiography. He underwent chest tube placement and was discharged five days later after returning to his baseline.

**Conclusion:**

Despite minimal data to support or refute the use of paramedic tele-ultrasound, this case highlights a unique opportunity to expand the use of telemedicine and ultrasound in community paramedicine to improve patient outcomes.

Population Health Research Capsule What do we already know about this clinical entity?

*Computed tomography is generally considered the gold standard for diagnosis of a pneumothorax.*

What makes this presentation of disease reportable?

*Using real-time video, an emergency physician diagnosed a pneumothorax by interpreting ultrasound images obtained by the paramedic on scene.*

What is the major learning point?

*The clinician’s telehealth consult with the paramedic on scene demonstrates a novel solution to diagnosing conditions where time to diagnosis affects outcomes.*

How might this improve emergency medicine practice?

*Emergency physicians will be able to diagnose and treat patients earlier, potentially leading to improved patient outcomes.*

## INTRODUCTION

With the emergence of telemedicine and ultrasound use combined with community paramedicine, health systems are finding novel ways to diagnose and treat both common and unusual diseases. However, evidence is still sparse regarding the use of telehealth and ultrasound by paramedics. This case report highlights a novel use of ultrasound by a community tele-paramedic program (CTP) to diagnose a pneumothorax in the prehospital setting and refer the patient to emergency care, leading to a good outcome.

A pneumothorax occurs when air accumulates between the parietal and visceral pleura inside the chest wall, causing the lung parenchyma to collapse.^1^ It can be either traumatic or atraumatic and is further classified as simple, tension, or open. Atraumatic pneumothorax is either primary, occurring without an inciting event, or secondary as in the setting of pulmonary disease.^1^ ^,^ ^2^ Traumatic pneumothorax is seen in 20% of blunt chest trauma, and up to 40% of penetrating trauma.^2^ Patients can be asymptomatic or present with shortness of breath, chest pain, tachycardia, decreased breath sounds, jugular venous distention, hypotension, cyanosis, and cardiac arrest.^1^

While computed tomography (CT) remains the gold standard of diagnosis (despite some debate), the diagnosis can also be made with ultrasound or with chest radiography.^3^ Ultrasound has a 94% sensitivity and up to a 100% specificity depending on the operator.^2^ Ultrasound findings include a loss of lung sliding in B-mode and the presence of a “barcode sign” on M-mode, signifying the loss of pleural movement.^2^ Chest radiography and CT findings include space between the pleura and chest wall.^3^ The differential diagnosis often includes cardiac tamponade, aortic dissection, rib fracture, acute coronary syndrome, pulmonary embolism, and pneumonia.^1^ Management depends on the severity of pneumothorax and can include the following: needle decompression, finger thoracostomy, pigtail thoracostomy, and large-bore tube thoracostomy.

The uniqueness of this case revolves around the method of diagnosis. The case patient was enrolled in a CTP program, which is part of a large, urban academic emergency department. Patients are jointly evaluated by community paramedics (CP) on scene as well as by an emergency physician (EP) over video conference. These paramedics underwent a one-hour long training session in lung point-of-care-ultrasound (POCUS) using the Butterfly iQ (Butterfly Network, Burlington, MA) connected to a mobile device. Images were obtained by the CP crew using the “lung” preset of the device, with the probe marker pointing superiorly, and interpreted in real-time by the EP over the telehealth platform. While some studies have reported the feasibility of teaching emergency medical services (EMS) professionals how to obtain and read POCUS images, there is a paucity of data regarding the regular use of POCUS in the prehospital setting and even fewer involving live image interpretation by an EP.^7^

## CASE REPORT

This case highlights an 86-year-old male who was being followed closely by the CTP program of an urban, academic hospital-based EMS system. His medical history included Parkinson disease, stroke, peripheral neuropathy, atrial fibrillation status-post multiple ablations, direct-current cardioversion, and implantation of a Watchman device, sick sinus syndrome status-post automatic implantable cardioverter defibrillator placement, congestive heart failure, chronic obstructive pulmonary disease (COPD), and frequent falls requiring multiple hospitalizations. He presented at a routine home visit complaining of left rib pain and shortness of breath after a fall two days prior. Of note, a month before this visit he had been hospitalized for a fall and declined the recommended subacute rehabilitation placement. The only home care services he received at the time were weekly CTP visits.

**Image 1.**
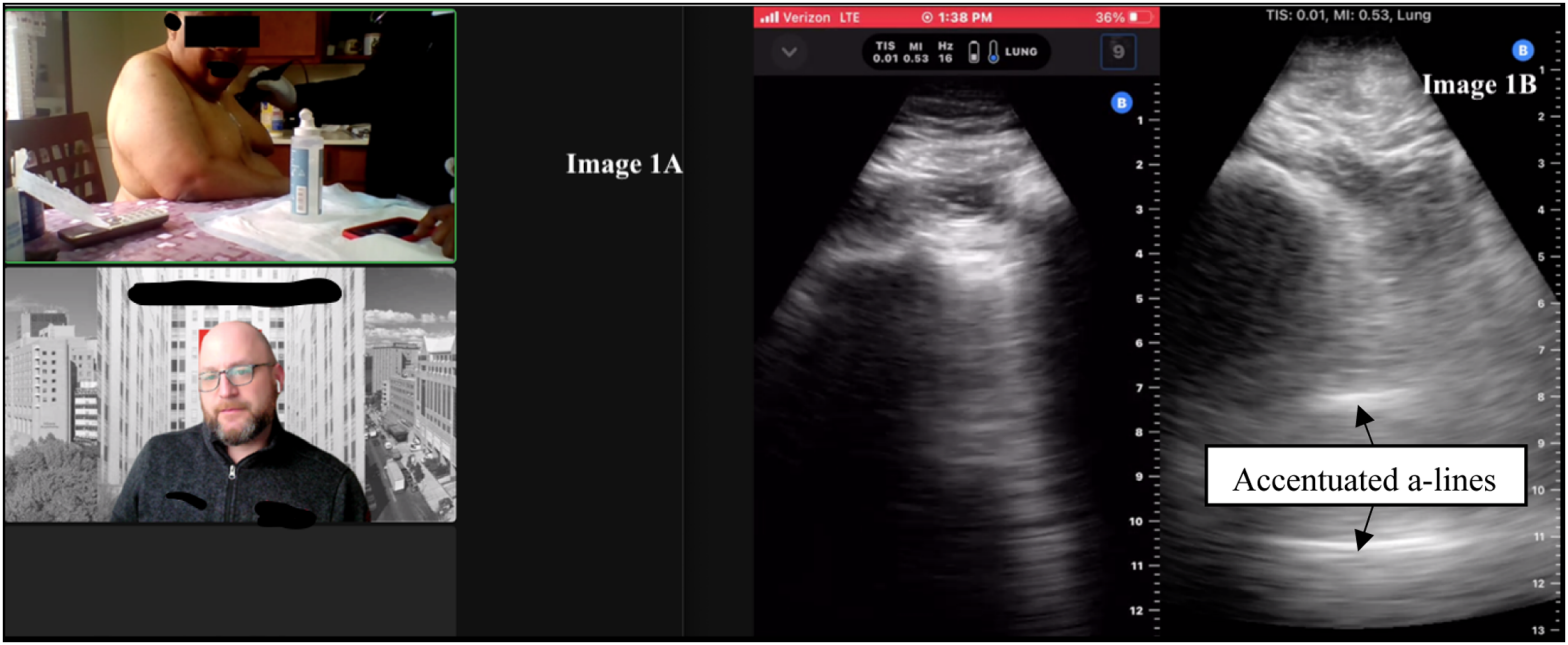
(A) Example of a community tele-paramedic visit with video conference and screen sharing using mobile ultrasound. The patient is seen at the top, and the emergency physician is seen on the bottom screen. (B) The case patient’s b-mode lung point-of-care ultrasound, showing accentuated a-lines, loss of b-lines, and in real-time video without lung sliding (not pictured here).

During his initial evaluation by paramedics he denied other complaints. Initial vitals were pulse 65 beats per minute, blood pressure 122/77 millimeters of mercury, pulse oximetry 100% on room air, temperature 36.6° Celsius, and respirations 19 breaths per minute. His physical exam was significant for lethargy, although he was easily arousable to voice; left-sided chest wall tenderness to palpation; bruising; crepitus; decreased left breath sounds; and bilateral lower extremity pitting edema. Paramedics obtained bilateral anterior views of the lung (Image 1a), and images were interpreted in real-time by the physician via a video telehealth platform. The EP noted the absence of lung sliding in the left inferior lung field (Image 1b).

**Image 2.**
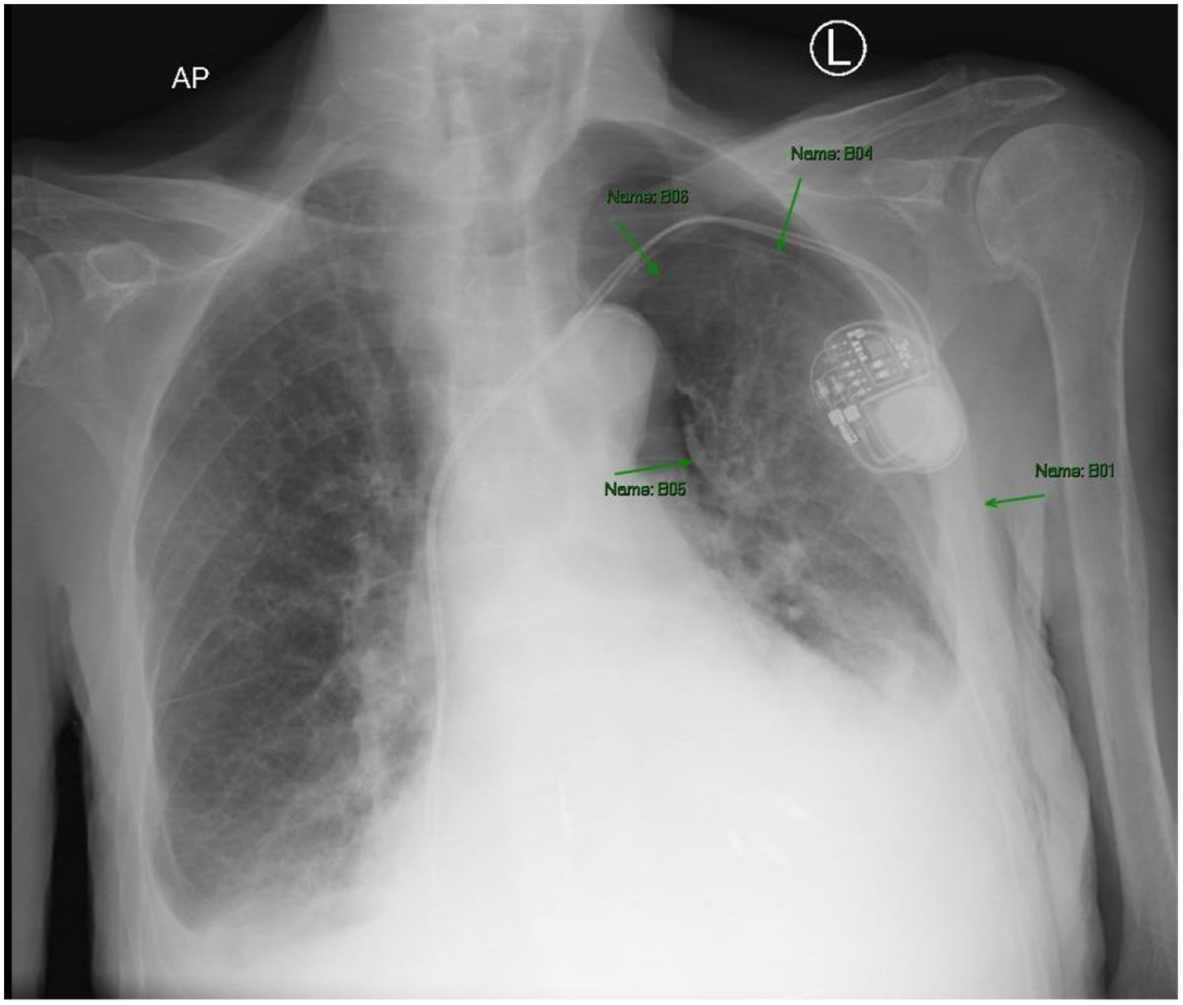
Chest radiograph with arrows pointing to left-sided moderate circumferential pneumothorax. There is a loss of symmetry, a visible lung border, and loss of lung marking superior to the lung border.

**Image 3.**
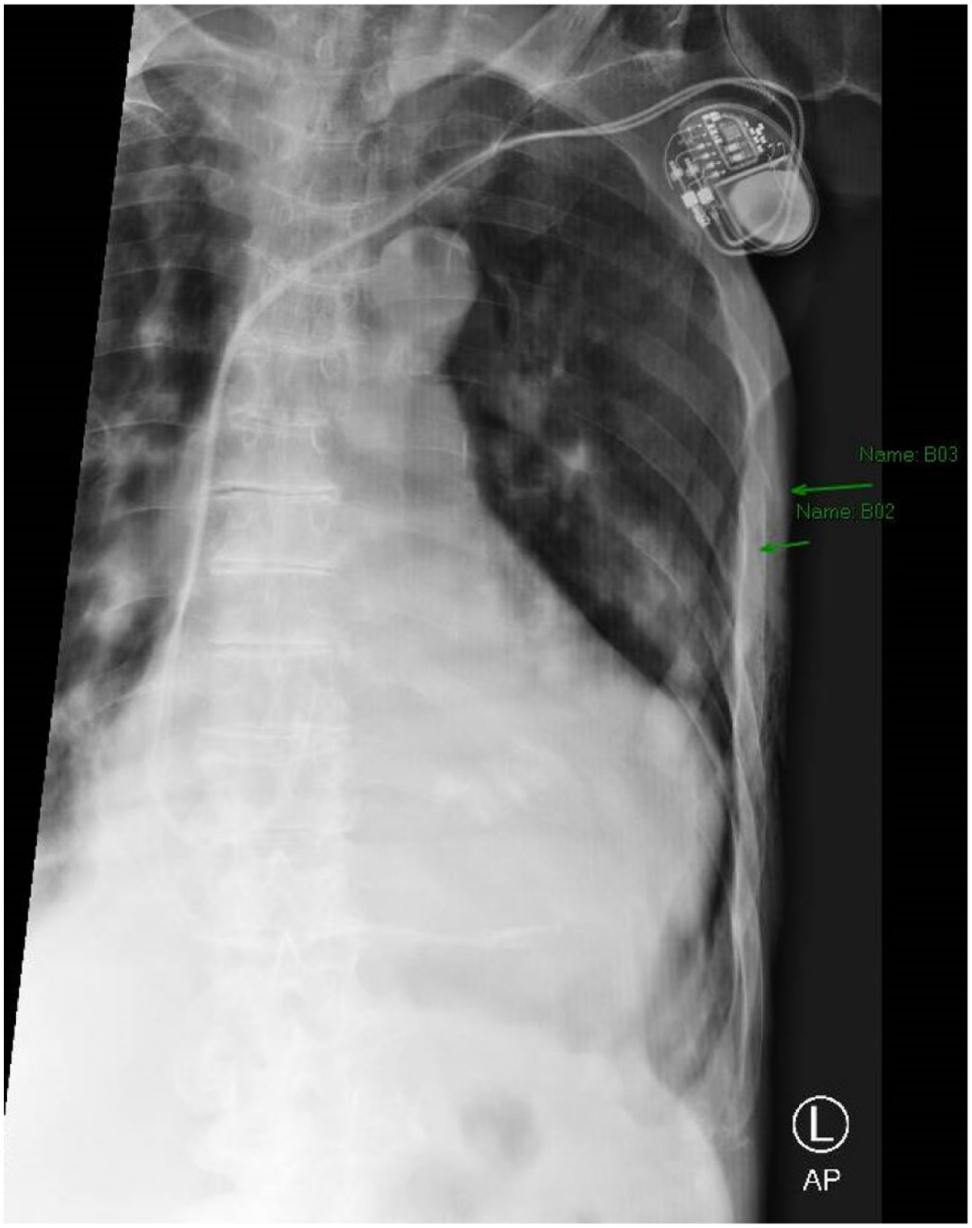
Magnified view of the chest radiograph demonstrating fractures of ribs five and six (arrows).

The patient was placed on 100% oxygen via non-rebreather mask and transported to a local emergency department (ED). Upon arrival at the ED he denied new complaints, and his vitals and physical exam were not significantly changed. His electrocardiogram (ECG) showed an atrial sensed paced rhythm consistent with prior ECGs. He had a chest radiograph (CXR) showing left fifth and sixth rib fractures with a moderate circumferential pneumothorax (Images 2 and 3).

He additionally had non-contrast CTs of the head and cervical spine showing interval resolution of prior subdural and subarachnoid hemorrhages without any acute findings. Serum complete blood count, chemistry panel, cardiac biomarkers, and coagulation profile were unremarkable. He was continued on 100% oxygen via a non-rebreather mask. Tube thoracostomy placement in the ED was deferred due to the patient’s clinical stability, presence of bilateral pleural effusions, and absence of a safe window to place a chest tube on ultrasound. The patient was admitted to the surgical service. On hospital day one, a repeat CXR showed an unchanged pneumothorax. He underwent an interventional radiology CT-guided pigtail thoracostomy placement. On hospital day three, the chest tube was clamped and subsequently removed. He was recommended for subacute rehabilitation; however, both the patient and family declined. He was discharged home on hospital day five with continued weekly follow up with the CTP program, visiting nurses, and home physical therapy.

The patient was seen with family by his primary care physician three days after hospital discharge. Home safety concerns were addressed, fall prevention teaching was given, and the patient chose a “do not resuscitate/do not intubate” status. He continues to be followed by the CTP program on a weekly basis, and unfortunately has had a subsequent admission for congestive heart failure exacerbation and two ED visits for falls.

## DISCUSSION

Although pneumothorax is not a rare condition and EPs are quite familiar with the diagnosis and treatment, clinicians must be aware of the increasing presence of virtual care and mobile integrated healthcare (MIH). The novelty surrounding this case lies in how the diagnosis was made. The paramedics are part of an EMS division under the department of emergency medicine at a large, urban, academic medical center. They are specially trained as CPs providing scheduled home visits to patients primarily with heart failure. Their role has expanded to include post-ED or post-hospital discharge follow-ups for conditions such as COPD and falls.

While there are national EMS standards on CP training, there are few to no standards regarding the training of EMS professionals in the use of POCUS.^5^ A scoping review regarding educational standards revealed less than 20 review articles, with a consensus showing little-to-no standardization and no consideration for level of training. However, some studies have shown that after a training program paramedics can accurately acquire and interpret lung POCUS for pneumothorax or tension pneumothorax with similar accuracy to EPs.^6^ ^,^ ^7^ It should be noted that most of these studies had assessments of simulated patients or patients with a known pneumothorax rather undifferentiated prehospital patients. The results of small pilot studies have suggested that prehospital POCUS performed by paramedics and interpreted via telehealth platform using cellular data has “good” to “very good” quality and that remote lung POCUS is feasible, although further research on reliability and clinical outcomes is needed.^8^ ^,^ ^9^

There is a paucity of quality data showing that immediate interpretation of lung POCUS leads to more rapid diagnosis, intervention, and better patient outcomes despite the potential of lung POCUS to positively impact immediate care. In this case we describe a unique method of diagnosis and rapid treatment leading to a positive patient outcome, which may have otherwise been missed leading to clinical decline, significant morbidity or even death if left untreated. This case highlights an opportunity for both EPs and EMS professionals to expand their scope of practice within the prehospital setting. The use of real-time interpretation by an EP over telemedicine (as compared to paramedic-only interpretation or asynchronous store and forward) affords the opportunity to guide image acquisition for less-experienced ultrasonographers and in cases of difficult patient windows, while providing additional clinical context to the EP reading the images. Overall, this has the potential to effectively triage patients to appropriate dispositions starting from very early on in their care.^10^

One limitation is the small body of literature evaluating whether early lung POCUS read by an EP improves clinical outcomes. This case report highlights the need for further implementation studies to better understand the risks and benefits of POCUS in the remote management of patient populations living with chronic illness such as heart failure and COPD where rapid evaluation and differentiation of the many causes of dyspnea at the patient’s side can be valuable for determining the most appropriate treatment and level of care.

With the case report we also sought to increase EP awareness of the possibility of prehospital use of POCUS. A needs assessment at our institution suggests that while EPs involved in the remote management of medically complex patients through MIH programs believe remote lung ultrasound is valuable, most were not aware it was available, safe, or effective. However, the data referred to above suggests that with the right training, paramedics are able to obtain ultrasound images. As emergency medicine expands to involve mobile integrated healthcare and virtual care, we believe EPs can expect to see that patient assessments by paramedics include POCUS images to interpret.

## CONCLUSION

This case of community tele-paramedicine use of lung POCUS read by an EP as a pneumothorax shows both the diagnostic diversity of pneumothorax and the feasibility of EMS professionals using POCUS to advance patient care. It is important to recognize a pneumothorax and treat it early to prevent progression to tension physiology. By partnering with EMS, we may be able to identify this diagnosis and initiate emergent treatment early on. Emergency physicians should be aware of the growing prevalence of prehospital ultrasound and its utility in the diagnosis of common lung pathology.
